# Differential susceptibility of reef-building corals to deoxygenation reveals remarkable hypoxia tolerance

**DOI:** 10.1038/s41598-021-01078-9

**Published:** 2021-11-30

**Authors:** Maggie D. Johnson, Sara D. Swaminathan, Emily N. Nixon, Valerie J. Paul, Andrew H. Altieri

**Affiliations:** 1 Smithsonian Marine Station, Fort Pierce, FL USA; 2 Tenenbaum Marine Observatories Network, Smithsonian Institution, Edgewater, MD USA; 3 Biology Department, Woods Hole Oceanographic Institution, Woods Hole, MA USA; 4 Department of Environmental Engineering Sciences, University of Florida, Gainesville, FL USA; 5 Present Address: Red Sea Research Center, King Abdullah University of Science and Technology, Thuwal, Saudi Arabia

**Keywords:** Marine biology, Ecology, Climate-change ecology

## Abstract

Ocean deoxygenation threatens the persistence of coastal ecosystems worldwide. Despite an increasing awareness that coastal deoxygenation impacts tropical habitats, there remains a paucity of empirical data on the effects of oxygen limitation on reef-building corals. To address this knowledge gap, we conducted laboratory experiments with ecologically important Caribbean corals *Acropora cervicornis* and *Orbicella faveolata*. We tested the effects of continuous exposure to conditions ranging from extreme deoxygenation to normoxia (~ 1.0 to 6.25 mg L^−1^ dissolved oxygen) on coral bleaching, photophysiology, and survival. Coral species demonstrated markedly different temporal resistance to deoxygenation, and within a species there were minimal genotype-specific treatment effects. *Acropora cervicornis* suffered tissue loss and mortality within a day of exposure to severe deoxygenation (~ 1.0 mg L^−1^), whereas *O. faveolata* remained unaffected after 11 days of continuous exposure to 1.0 mg L^−1^. Intermediate deoxygenation treatments (~ 2.25 mg L^−1^, ~ 4.25 mg L^−1^) elicited minimal responses in both species, indicating a low oxygen threshold for coral mortality and coral resilience to oxygen concentrations that are lethal for other marine organisms. These findings demonstrate the potential for variability in species-specific hypoxia thresholds, which has important implications for our ability to predict how coral reefs may be affected as ocean deoxygenation intensifies. With deoxygenation emerging as a critical threat to tropical habitats, there is an urgent need to incorporate deoxygenation into coral reef research, management, and action plans to facilitate better stewardship of coral reefs in an era of rapid environmental change.

## Introduction

Ocean deoxygenation is escalating in both the open ocean and coastal ecosystems due to warming and local nutrient pollution, and is now recognized as one of the leading environmental threats to the persistence of marine ecosystems and the services they provide^1–3^. Deoxygenation manifests in nearshore habitats as acute episodes (i.e., hypoxic events) during which resident organisms experience depleted oxygen levels for prolonged periods^2^. The occurrence of these events is increasing in both frequency and severity worldwide, and coupled with intensifying global deoxygenation, has dire consequences for marine taxa^4–7^. In temperate ecosystems, catastrophic loss of key taxa and habitat due to hypoxia following exposure to deoxygenation events has led to the decline and loss of ecosystem function^8,9^ and economic value^10^.

While impacts of deoxygenation have been a focus of study in the open ocean (e.g., oxygen minimum zones) and temperate coastal habitats (e.g., estuaries, coastal seas) for decades^2,4,6^, the importance of similar trends has only recently emerged in the tropics where the threat deoxygenation poses to coral reefs is becoming increasingly apparent (reviewed in^11^ and^12^). Approximately 13% of all coral reef habitat is at elevated risk to deoxygenation events, and that number is likely underestimated by an order of magnitude^13^. Such events can cause mass mortality and dramatically impact reef ecosystems and the goods and services they provide to coastal communities^14^. With this growing awareness of the role of deoxygenation in the future of coral reefs, the gaps in our knowledge base of hypoxia impacts on reef corals have become clear and require urgent action^12,15^.

Episodes of hypoxia related to deoxygenation associated with stratification, eutrophication, coral spawn slicks, or algal blooms have been implicated in bleaching, mass mortality, and formation of dead zones on coral reefs across the globe, from the Caribbean^13,16^ and Gulf of Mexico^17,18^ to the Great Barrier Reef^19^ and Indian Ocean^20^. Of the events that have been documented, the organismal impacts of hypoxia scale up to ecosystem-wide consequences that include loss of benthic biodiversity and live habitat^13,21^, as well as shifts in reef-associated microbial assemblages^17,21^. In some instances, the alterations in community structure resulting from hypoxia-induced, mass coral mortality can persist for years after the initial occurrence and permanently alter the reef habitat^21^. Despite the postmortem documentation of these catastrophic events, the link between acute deoxygenation and coral mortality on coral reefs has been conclusively reported in just a few cases (reviewed in^12^ and^22^). This dearth of in situ evidence may be because acute episodes are generally fleeting, and typically go unnoticed and undocumented, leading to an underestimation of deoxygenation impacts in the tropics. In addition, mass mortality events due to hypoxia may be uncommon on reefs because the frequent and predictable occurrence of hypoxia in tropical ecosystems may have led corals to evolve mechanisms of resilience, as recently suggested^23^. This may explain why some corals appear to persist through deoxygenation events, even in notable occasions of catastrophic mortality that have garnered attention^13,24^. The occurrence of species-specific differential susceptibilities could lead to the loss of intolerant species and a shift in community assemblages towards stress-tolerant species, rather than complete loss of live coral^25^.

More thoroughly studied stressors have revealed remarkable plasticity in coral responses to environmental stress^26^, including heightened tolerance to thermal stress^27,28^, acidification^29^ and disease susceptibility^30^. Tolerances can vary at the level of species and at the level of genotype of the coral host or symbiont^31–33^. Does this same variation in tolerance based on coral species and genotypes apply to deoxygenation stress as well? Comparisons across studies with single species, varying methods, and uncontrolled or poorly documented hypoxia in the field suggest that such variation likely exists among corals (reviewed in^12,22^. Comparative tests under controlled conditions are required to elucidate the varied and nuanced responses of tropical corals to hypoxia, yet to date, only a handful of such studies have been conducted^13,34–36^. For example, Alderice et al. (2020) identified species-specific phenotypic responses to deoxygenation in two acroporids and divergent hypoxia tolerances^34^. Indeed, identifying species and genotypes with differential susceptibilities to common environmental stressors is an essential step towards understanding and better predicting how reef habitats will shift in response to escalating local and global threats, and ultimately can be used to inform strategies to protect, manage, and restore important reef habitat^37,38^.

**Figure 1:**
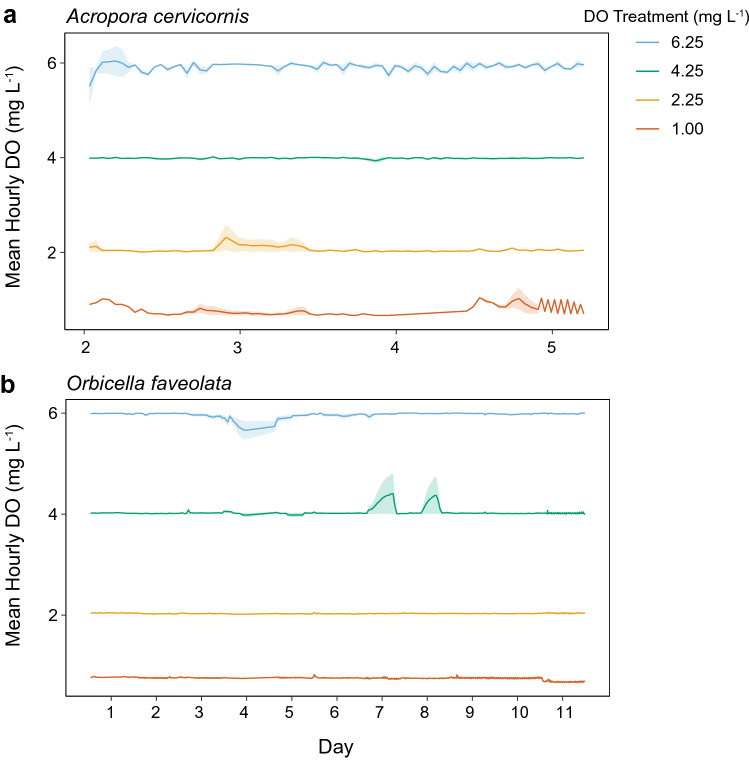
Dissolved oxygen treatment conditions. Mean hourly dissolved oxygen (DO) concentrations in treatment tanks (n = 3), with SD shown as shaded areas around the mean. Oxygen treatments were maintained through an oxygen-feedback, with continuous monitoring every 15 s for the duration of each experiment. (**a**) *Acropora cervicornis* was exposed to treatment conditions for 5 days and (**b**) *Orbicella faveolata* for 11 days. Day 1 represents the start of the experiment and first day of biological measurements.

Here we explored the potential for differential susceptibility to deoxygenation among common Caribbean corals with known genotypes. We conducted laboratory experiments with two coral species, *Acropora cervicornis* and *Orbicella faveolata*, designated as IUCN species of concern that have historically been dominant reef-builders in the Caribbean. Both species were exposed to persistent deoxygenation at four levels ranging from severe deoxygenation at ~ 1.0 mg L^−1^ to normoxia at ~ 6.25 mg L^-1^ (Fig. 1, Table 1). Within this experimental framework our objectives were (1) to quantify the effects of deoxygenation on coral photophysiology, bleaching, and mortality, (2) to explore the potential for species-specific tolerances or sensitivities to deoxygenation, (3) to identify whether genotypes within a species vary in their response to deoxygenation, and (4) to test whether these responses followed a linear relationship with oxygen availability. Our results provide insight to species-specific hypoxia thresholds in ecologically important Caribbean reef-building corals, and can be used to better predict the impacts of escalating deoxygenation on reef communities.

**Table 1:** Mean physical parameters (SD) from daily discrete measurements.

Species	Treat	DO (mg L^−1^)	Temp (ºC)	pH	Sal	PAR
*O. faveolata*	6.25	6.25 (0.06)	26.6 (0.02)	7.99 (0.05)	35.11 (0.16)	294 (16)
	4.25	4.24 (0.04)	26.6 (0.03)	8.00 (0.05)	35.07 (0.10)	310 (20)
	2.25	2.27 (0.03)	26.5 (0.02)	8.07 (0.04)	35.07 (0.10)	322 (40)
	1.00	0.94 (0.07)	26.6 (0.02)	8.24 (0.05)	35.08 (0.09)	296 (14)
*A. cervicornis*	6.25	6.37 (0.03)	26.4 (0.09)	7.91 (0.03)	35.20 (0.12)	319
	4.25	4.26 (0.06)	26.4 (0.11)	7.86 (0.04)	35.20 (0.09)	317
	2.25	2.33 (0.06)	26.2 (0.12)	7.98 (0.03)	35.22 (0.15)	312
	1.00	0.86 (0.05)	26.3 (0.23)	8.10 (0.02)	35.35 (0.23)	310

All measurements, were calculated as overall treatment means for the duration of each experiment (*O. faveolata*: n = 11 days; *A. cervicornis*: n = 5 days). PAR was measured on 3 different days for *O. faveolata* and on 1 day for *A. cervicornis*. pH is on the NBS scale, salinity is in practical salinity units, and PAR is in µmol photons m^−2^ s^−1^.

## Results

### Coral condition

**Figure 2:**
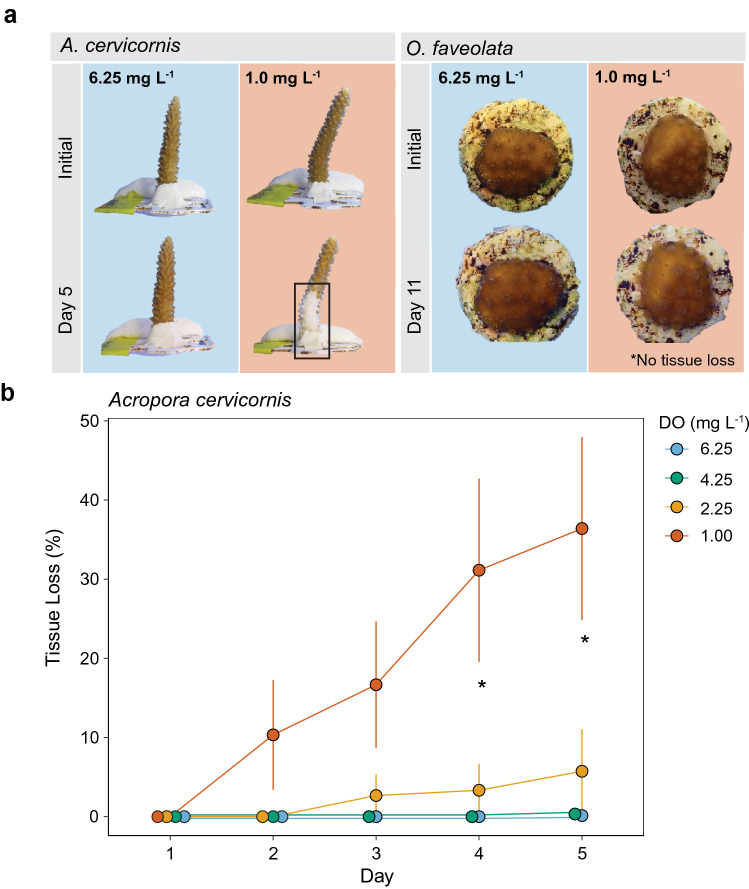
Tissue loss under deoxygenation. (**a**) Representative photos of coral fragments in the 6.25 and 1.00 mg L^−1^ dissolved oxygen (DO) treatments at the start and end of experiments. Fragments were monitored for visual signs of coral bleaching or tissue loss (black box) daily. (**b**) Mean ± SE percent tissue loss of *A. cervicornis* fragments over the duration of the study (n = 3). Day 1 represents measurements made on the first day of the experiment. Asterisks indicate significant differences (*P* < 0.05) between the 1.00 mg L^−1^ treatment and all other treatments on a given day.

To evaluate the effect of deoxygenation on overall coral condition, we monitored tissue loss as a percentage per fragment every day during experiments. There was an increase in tissue loss over time in *A. cervicornis* fragments, with a significant interaction between day and treatment (*P* < 0.001) (Fig. 2, Table 2). There were no significant interactions between treatment and genotype, genotype and day, or the three-way interaction among all factors (Table 2). Tissue loss was first apparent in *A. cervicornis* on Day 2 in the severe deoxygenation treatment, and was significantly higher in only the 1.0 mg L^−1^ treatment relative to all others on Days 4 and 5 (Tukey’s post hoc, *P* < 0.05 each day) (Fig. 2b). No tissue loss or partial mortality were observed in *A. cervicornis* in the 4.25 and 6.25 mg L^−1^ dissolved oxygen treatments (Fig. 2b). There were no observable changes in tissue condition of *O. faveolata* throughout the 11-day experiment in any treatment (Fig. 2a).

**Table 2:** Results of the linear mixed effects model evaluating the effect of fixed factors day, treatment, and genotype on tissue loss in *A. cervicornis*.

Species	Factor	Tissue Loss	
		F	*P*
*A. cervicornis*	Day	6.336	** < 0.001**
	Treatment	2.229	0.162
	Genotype	1.411	0.253
	Day*Treat	4.305	** < 0.001**
	Day*Geno	0.762	0.726
	Treat*Geno	1.063	0.421
	Day*Treat*Geno	0.648	0.959

Significance at *P* < 0.05 is noted in bold.

### PAM fluorometry

**Figure 3:**
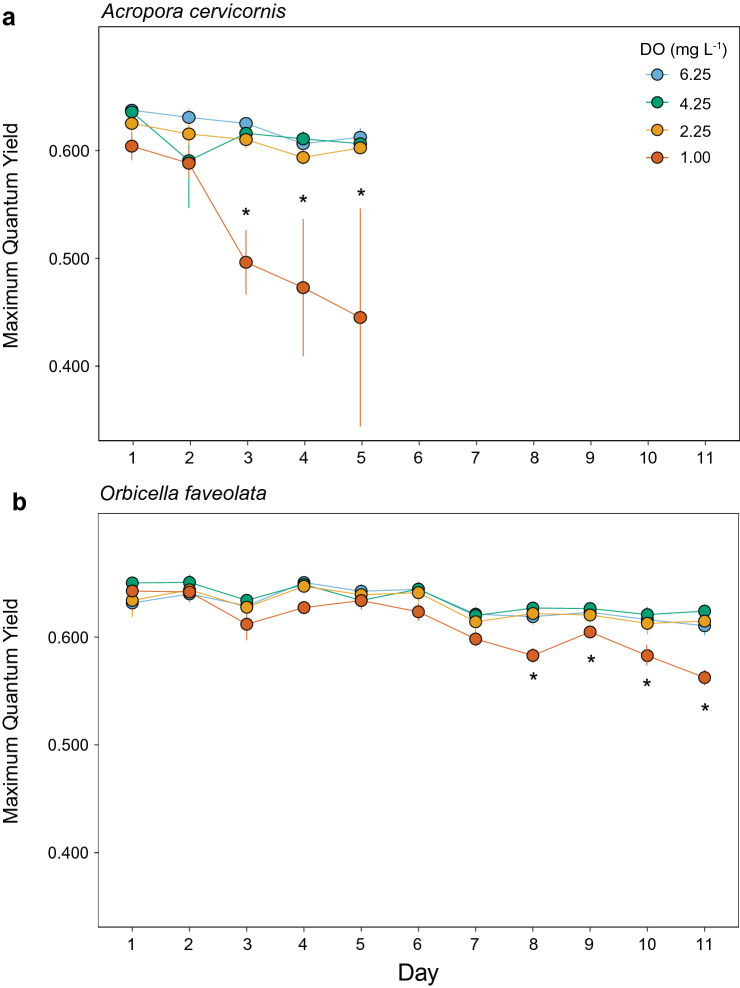
Maximum quantum yield under deoxygenation. (**a**) Mean ± SE quantum yield for *Acropora cervicornis* and (**b**) *Orbicella faveolata* for the duration each experiment (n = 3). Corals were measured prior to dawn for maximum quantum yield (F_v_/F_m_). Asterisks indicate significant differences (*P* < 0.05) between the 1.00 mg L^−1^ treatment and all other treatments on a given day.

To understand the effect of deoxygenation on coral performance, we used a pulse amplitude modulated (PAM) fluorometer to assess photophysiology of each coral fragment daily for the duration of experiments. There were no significant three-way interactions among treatment, day, and genotype for maximum quantum yield (F_v_/F_m_) in either species (Table 3). F_v_/F_m_ was affected by exposure to only severe deoxygenation (1.0 mg L^−1^) in both coral species, but the magnitude of response and time-scale of impact differed by species (Fig. 3). In the 1.0 mg L^−1^ treatment, deoxygenation had an earlier and more severe negative effect on F_v_/F_m_ in *A. cervicornis* than in *O. faveolata* (Fig. 3).

**Table 3:** Results of linear mixed effects models evaluating the effect of fixed factors day, treatment, and genotype on maximum quantum yield (F_v_/F_m_).

Species	Factor	Maximum Quantum Yield	
		F	*P*
*O. faveolata*	Day	64.308	** < 0.001**
	Treatment	3.047	**0.038**
	Genotype	2.240	0.065
	Day*Treat	5.634	** < 0.001**
	Day*Geno	1.735	**0.002**
	Treat*Geno	0.554	0.894
	Day*Treat*Geno	0.826	0.918
*A. cervicornis*	Day	7.114	** < 0.001**
	Treatment	4.378	**0.042**
	Genotype	1.192	0.333
	Day*Treat	3.101	** < 0.001**
	Day*Geno	1.286	0.212
	Treat*Geno	1.378	0.226
	Day*Treat*Geno	0.875	0.699

Significance at *P* < 0.05 is noted in bold.

In *A. cervicornis*, there was a significant interactive effect of treatment and day on F_v_/F_m_ (*P* < 0.001; Table 3), where the decline in F_v_/F_m_ intensified with longer exposure to treatment conditions (Fig. 3a). The oxygen treatment effect was driven by significant declines in F_v_/F_m_ in the 1.0 mg L^−1^ treatment, relative to the three other DO treatments. F_v_/F_m_ in *A. cervicornis* began to decline after just two days of exposure to 1.0 mg L^−1^ conditions, and remained significantly lower in the 1.0 mg L^−1^ treatment than all others from Day 3 until the end of the experiment (Tukey’s, *P* < 0.05) (Fig. 3a). There were no significant interactions among other factors, and no genotype-specific responses (Fig. S1, Table 3).

In *O. faveolata*, there was a significant interactive effect of treatment and day on F_v_/F_m_ (*P* < 0.001, Table 3), with an intensifying effect of exposure in only the 1.0 mg L^−1^ treatment (Fig. 3b). Notably, F_v_/F_m_ began to decline in the 1.0 mg L^−1^ treatment after seven days of exposure, compared to two days in *A. cervicornis*, and continued to decline until the end of the experiment (Tukey’s posthoc, *P* < 0.05). However, *O. faveolata* F_v_/F_m_ declined by just 12% in the 1.0 mg L^−1^ treatment over the 11-day experiment, with no change over time in the other three DO treatments (Fig. 3b). There was also a significant interactive effect of genotype and day on F_v_/F_m_ in *O. faveolata* (*P* = 0.002; Table 3), which was largely driven by overall lower F_v_/F_m_ for genotype F132 in all treatments, except the 1.0 mg L^−1^ treatment in which F132 was similar to all other genotypes by the end (Fig. S2).

### Symbiont density

**Figure 4:**
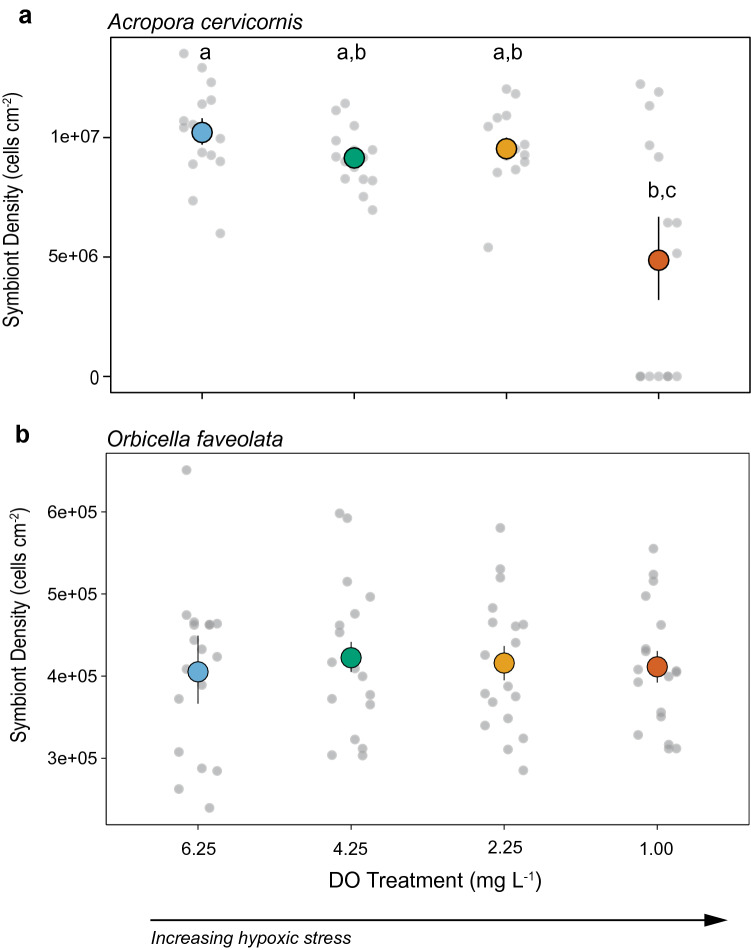
Coral symbiont densities under deoxygenation. (**a**) Mean ± SE symbiont densities at the end of the 5-day experiment for *Acropora cervicornis* and (**b**) the 11-day experiment for *Orbicella faveolata* (n = 3). The grey circles show individual data points and colored circles represent means across all genotypes within a treatment. Treatments with different lower-case letters are statistically different at *P* < 0.05.

To quantify coral condition and bleaching we sampled coral fragments at the end of the experiment for densities of endosymbiotic algae (Symbiodiniaceae). There was a significant effect of treatment on symbiont densities in *A. cervicornis* (*P* = 0.047), and no effect of genotype (*P* = 0.279) or interaction between treatment and genotype (*P* = 0.667; Fig. 4, Table 4). Symbiont densities were significantly lower in the 1.0 mg L^−1^ treatment relative to the normoxic treatment (6.25 mg L^−1^) (Tukey’s post hoc, *P* < 0.05) (Fig. 4a).

**Table 4:** Results of linear mixed effects models evaluating the effect of fixed factors treatment, and genotype on coral symbiont density.

Species	Factor	Symbiont density	
		F	*P*
*O. faveolata*	Treatment	0.091	0.963
	Genotype	13.808	** < 0.001**
	Treat*Geno	1.059	0.421
*A. cervicornis*	Treatment	4.098	**0.047**
	Genotype	1.270	0.279
	Treat*Geno	0.785	0.667

Significance at *P* < 0.05 is noted in bold.

There were no significant effects of oxygen treatment on *O. faveolata* symbiont densities (Fig. 4b), and no significant interactive effects (Table 4). However, symbiont densities varied significantly by genotype (*P* < 0.001) and were higher in genotypes F8 and F27 than the other four genotypes (Tukey’s post hoc, *P* < 0.05) (Fig. S3).

## Discussion

Here we reveal how two ecologically important reef-building Caribbean coral species exhibited vastly different responses to oxygen depletion. *Acropora cervicornis* was highly sensitive to deoxygenation, and suffered bleaching, tissue loss, and mortality under severe deoxygenation (1.0 mg L^−1^ dissolved oxygen), with symptoms beginning after just one day of exposure. Half of all *A. cervicornis* in the 1.0 mg L^−1^ treatment showed signs of partial or full mortality within five days. Maximum quantum yield (F_v_/F_m_) and symbiont densities also were negatively affected by severe deoxygenation. Conversely, after 11 days of exposure to the same treatment conditions, *O. faveolata* showed no visual signs of bleaching, tissue loss, or partial mortality in any oxygen treatment. *Orbicella faveolata* F_v_/F_m_ began to decline after a week of exposure to severe deoxygenation, but the magnitude of this response was small. Within each species, there were no significant genotype-specific responses to deoxygenation. Collectively, our results show that some corals can have drastically different hypoxia thresholds, and that even the more sensitive coral in our experiment, *A. cervicornis*, withstood prolonged exposure to levels of deoxygenation (e.g., 2.25 mg L^−1^) that have been found to be lethal for other marine taxa^2,12^.

Among the most notable responses in our study were the rapid bleaching and tissue loss in *A. cervicornis* under severe deoxygenation (1.0 mg L^−1^) and the lack of response in *O. faveolata*. These divergent effects of deoxygenation indicate different hypoxia-thresholds between the two species, a pattern that has also been observed in species of Indo-Pacific acroporids^34^. To date, the few controlled studies to have explored coral responses to oxygen stress have shown variability in hypoxia-thresholds among coral species^13,34–36^. For example, Alderice et al. (2020) found that *Acropora selago* bleached under deoxygenation levels of 2 mg L^−1^ in under 12 h, while the closely related *Acropora tenuis* did not^34^, and Haas et al. (2013) found that *Acropora yongei* showed signs of bleaching following exposure to 2–4 mg L^−1^ ^35^. In the present study, the hypoxia threshold for *A. cervicornis* appears to be even lower. *Acropora cervicornis* individuals showed signs of stress, indicated by bleaching, that began within 1–2 days of exposure to severe deoxygenation (1.0 mg L^−1^) only, and not in response to conditions of 2.25 mg L^−1^ or higher. In addition, *O. faveolata* was tolerant to even the most severe deoxygenation treatment.

The presence of species-specific variability in deoxygenation responses was not unexpected, considering that corals frequently show species level variation in bleaching susceptibility^32,39^ and tolerance to other environmental conditions including pH^25,40^, nutrient enrichment^41^, and warming^42,43^. However, the magnitude of differential effects and temporal scale of response between *A. cervicornis* and *O. faveolata* was unexpected. Because the species used in this study were sourced from different (but nearby) coral nurseries, it is worth considering how history of exposure to environmental conditions could have influenced the observed patterns. On average, the ambient conditions of the in situ nursery are well within the tolerance limits of each species (Fig. S4), there are high turnover rates and consistent flushing, and the magnitude of daily variability in DO in these open systems is minimal (Fig. S4). The immediate environmental history, prior to transport to the Smithsonian Marine Station, is not known in detail for *O. faveolata*. However, the ex situ coral nursery where fragments were maintained is an open system with continuous flow-through of seawater. Consistent flow rates, combined with continuous bubbling by air stones, indicates the oxygen conditions were likely at 100% saturation with the atmosphere. Regardless of similarities in environmental conditions at the source coral nurseries, environmental history can influence subsequent responses to environmental stressors^44^. To reduce the potential effects of environmental history on treatment responses, we maintained each species under similar acclimation conditions in the wet lab facilities at the Smithsonian Marine Station for 4–6 weeks prior to the experiments. This period of time is generally sufficient to mitigate the effects of the immediate environmental history on subsequent responses to stress^45^. The observations on species-effects we observe here should be interpreted with these caveats in mind.

Although the potential for different environmental exposure in the coral nurseries cannot be fully disentangled from species effects, the magnitude of the divergence in the response of *O. faveolata* and *A. cervicornis* to deoxygenation, along with the extended acclimation period in the lab, suggests a species-level response. This is further supported by analogous studies with different stressors. For example, heightened sensitivity of *A. cervicornis* to environmental stress is supported by a suite of studies that have shown some acroporids to be highly susceptible to warming^39^, bleaching^28^, disease^46^, and deoxygenation^34,35^. Conversely, we found that *O. faveolata* was largely tolerant to deoxygenation, and persisted for more than a week before showing minimal impacts on only maximum quantum yield. Literature on general responses of *O. faveolata* to environmental stressors are more limited and mixed, and typically centered around responses to thermal stress. For example, in response to warming *O. faveolata* has demonstrated bleaching resistance^47^, increased susceptibility to repeated bleaching^48^, and heightened capacity to recover following repeated bleaching^49^. Our results indicate that *O. faveolata* is largely tolerant to hypoxia. Although sensitivity to other environmental stressors can sometimes predict coral susceptibility to deoxygenation, as with *A. cervicornis*, it may not inherently apply to other species, such as *O. faveolata*, where the literature reveals mixed responses.

The tolerance of *O. faveolata* to deoxygenation provides promising evidence for the capacity of some corals to withstand oxygen deprivation for extended periods of time. We exposed corals to static deoxygenation treatments, whereas oxygen dynamics on coral reefs can be highly variable and potentially influence organismal responses to subsequent stress^44^. Therefore, the extended duration of *O. faveolata* tolerance to static deoxygenation may be most representative of conditions that can occur during acute deoxygenation events rather than diel cycling. The duration of prolonged exposure to oxygen deprivation during acute events is largely unknown for the tropics. However, temperate studies show that hypoxic episodes can persist for days to weeks^4^, during which time resident organisms are consistently exposed to anoxia or severe deoxygenation. Survival of only the relatively tolerant taxa through an acute event could lead to shifts in coral assemblages towards dominance of tolerant species, rather than outright loss of coral populations, a trend that has been observed on Caribbean reefs following acute deoxygenation events^13,21^. Variation in hypoxia sensitivities and shifts towards more tolerant species suggest that reefs have the capacity to persist when challenged with deoxygenation, although shifts such as those described above can result in a change in the dominant morphology of reef-building corals away from branching corals and impact ecological functioning^50^.

Intraspecific variability is another factor that potentially contributes to organismal stress responses, and a goal of our study was to evaluate the role of genotype in determining coral hypoxia thresholds. We found no clear genotypic responses to deoxygenation, although we did detect a genotype difference in baseline symbiont densities. Our ability to detect genotype-specific responses may have been impeded by replication within a genotype that was constrained by our mesocosm array (n = 3 per treatment), a limitation that has been identified in comparable studies^51^. Furthermore, the co-mingling of genotypes within replicate treatment tanks should be taken into consideration. Although the colonies were > 10 cm apart, the different genotypes within a species were not independent of one another within a tank. Due to these constraints we are not able to fully resolve the role of genotype in coral responses to deoxygenation, and the apparent lack of a genotypic response should be interpreted cautiously and explored in greater detail with additional genotypes and replication in future studies.

An additional caveat to our study is that we did not have the capacity to manipulate pH while maintaining DO levels in treatments via nitrogen bubbling. As a result, pH tended to be slightly higher in the deoxygenation treatments than the normoxic treatments (Tables 1, 2), and this should be considered as a potential factor contributing to coral responses. The most severe deoxygenation treatment had higher pH (i.e., less acidic), while the normoxic treatments tended to have lower pH (i.e., more acidic). Because potential stress associated with changes in DO and pH were in opposing directions (oxygen was more stressful while pH was less stressful and vice versa), and our experiments ran for a shorter interval than typical studies involving acidification, it is unlikely that pH was a major driver in the dramatic responses to severe deoxygenation we document here. Future work could address this issue by incorporating CO_2_ manipulation into the experimental design to offset changes in pH due to nitrogen bubbling.

Here we show that some corals possess a remarkable capacity for resilience to severe and intermediate levels of deoxygenation, while others are more sensitive. Our findings contribute to the growing body of work illustrating variability in hypoxia-tolerances among reef-building corals, and emphasize the importance of evaluating susceptibility to deoxygenation when considering the impacts of environmental change on ecologically valuable reef taxa. Quantifying hypoxia-thresholds in reef-building corals provides valuable insight to how coral reefs may change in the coming decades as deoxygenation intensifies and acute events become more frequent, and is an area of research that remains ripe for further work. To fill knowledge gaps, future work should focus on: 1) elucidating hypoxia-thresholds of foundational reef taxa and evaluating species-specific tolerances to deoxygenation, 2) identifying the role of genotypic variation in shaping coral hypoxia-tolerances using independent and sufficient replication, 3) continuing to uncover the molecular basis of differential sensitivities to deoxygenation, and 4) identifying how organismal impacts of deoxygenation influence ecosystem dynamics and community trajectories. Coral reefs are facing the triple threat of exposure to warming, ocean acidification, and deoxygenation, and how the cumulative impacts of these global stressors manifest at the local scale will be contingent on the composition of community assemblages and their capacity for resistance and resilience under escalating environmental stress.

## Methods

### Coral collection and laboratory acclimation

Corals were obtained from the nursery at Mote Marine Laboratory’s Elizabeth Moore International Center for Coral Reef Research & Restoration. *Acropora cervicornis* fragments (apical tips ~ 5–6 cm in length) were obtained from Mote’s in situ nursery and mounted to a plastic base with marine epoxy (Instant Ocean Holdfast). The *A. cervicornis* genotypes used in this study were Mote Marine Laboratory genotype numbers 7, 31, 50, 57, and 70 (n = 12 ramets from each of the 5 genets). The coral *O. faveolata* was obtained from Mote’s ex situ nursery as fragments (~ 3–4 cm^2^) attached to individual carbonate plugs. Six genotypes of *O. faveolata* were used in deoxygenation treatments from genotype numbers F3A, F8, F27, F61, F125, and F132 (n = 12 ramets from each of the 6 genets). The immediate environmental conditions at the ex situ coral nursery are not known in detail, however the corals were maintained in an open, flow-through system with high flow rates and continuous bubbling with air stones.

Corals were transported to the wet lab facilities at the Smithsonian Marine Station (SMS) in Fort Pierce, FL and experiments were conducted between October and December 2019. Corals were acclimated to laboratory conditions for 4–6 weeks before exposure to deoxygenation treatments. During this period, corals were kept in an indoor, closed seawater system. Holding tanks contained ~ 570 L of seawater that was recirculated through a sump containing rigorous aeration (ambient air) and a heater/chiller that maintained the temperature at ~ 27 ºC (see Table S3 for all parameters). Water was pumped from the sump through a UV sterilizer (Coralife) and then back into the holding tank. Water flow and circulation was maintained by two aquarium pumps (AquaTop MaxFlow MCP-5), and yielded a full water exchange through the sump ~ 8.5 times per hour. Light was provided by LED aquarium lights (HQD) with a maximum photosynthetically active radiation (PAR) of ~ 300 µmol photon m^−2^ s^−1^. Full water changes were conducted weekly, and non-living surfaces on the coral fragments (e.g., plastic or carbonate bases) were cleaned at least once a week to reduce algal growth. Conditions within the acclimation tanks closely matched the ambient (i.e., normoxic) conditions at the collection locations and in the oxygen treatments (Table S3, Fig. S4).

### Field dissolved oxygen conditions

Oxygen concentrations were monitored at Mote Marine Laboratory’s in situ nursery (24.56 latitude, − 81.40 longitude) at the depth of coral outplants (~ 5–6 m) to parameterize oxygen conditions used in laboratory experiments. A dissolved oxygen sensor (miniDot, PMEL) was calibrated according to manufacturer protocols and attached to the benthos adjacent to coral outplants. Dissolved oxygen and temperature were logged every ten minutes for the duration of deployment. Hourly and daily average DO concentrations are presented for one month encompassing the approximate time of coral retrievals (Fig. S4). The 6.25 mg L^−1^ treatment in the laboratory experiments simulates the average normoxic conditions at the site of collection (6.18 ± 0.10, SD), while the most severe deoxygenation treatment (1.00 mg L^−1^) simulated extreme oxygen depletion that has occurred during acute deoxygenation events on coral reefs^21,22,52^ (Fig. S4). The two intermediate deoxygenation treatments represented incremental increases by ~ 2 mg L^−1^ to capture coral responses over a full range of oxygen conditions (Fig. S4).

### Experimental design

Deoxygenation experiments were conducted in the wet lab facilities at SMS using a mesocosm array consisting of 12 tanks (50 L, AquaLogic Systems), with each tank functioning as a closed system with fully independent temperature and oxygen control. Targeted oxygen levels for treatments were 1.00, 2.25, 4.25, and 6.25 mg L^−1^ DO, with three independent tank replicates for each oxygen level. Oxygen treatments are referred to by the targeted DO concentrations. Species were run in separate, sequential experiments, and genotypes within a species were co-mingled in treatment tanks with one replicate of each genotype per tank.

Oxygen concentrations were maintained in each independent tank by bubbling seawater with nitrogen gas and ambient air, with gas injection controlled through a DO feedback and solenoid valves (Neptune Systems). Oxygen levels were monitored every 15 s in each tank by an OxyGuard DO probe connected to an aquarium controller (Neptune Systems, Apex Aquacontroller), which opened or closed the respective solenoid valves to add nitrogen or air in order to maintain the programmed treatment conditions. Oxygen probes were calibrated at the start of experiments, and then again after five days, following the manufacturer's protocol.

Temperature control was maintained in treatment aquaria through an independent heating/chilling loop in each tank and monitored by AquaLogic temperature probes (sensu^53^). The average temperature at the collection site in the month prior to collection was 29.01 ± 0.62 ℃ (SD) (Fig. S4), which represents seasonally warm temperatures of the Florida Keys^54,55^. To reduce the potential confounding effects of temperature on responses to deoxygenation, we acclimated corals to 27 ℃ during the holding period, and conducted experiments at ~ 27 ℃. This represents the average annual temperature at the collection site in the Florida Keys, is the temperature that *O. faveolata* fragments were maintained at in the ex situ nursery prior to collection (27.0 ± 0.62 ℃), and is commonly used as the ambient temperature for corals from this location^54,56^. Each tank contained an additional probe (Neptune Systems) that logged temperature every 10 s for the duration of the experiment. In addition to continuous monitoring of DO and temperature, discrete measurements were taken each day for DO, temperature, pH, and salinity with a handheld multi-parameter water quality meter (YSI ProDSS with optical DO sensor) (Tables 1, 2, Tables S1, S2). The YSI DO probe was calibrated at the start of every day following manufacturer protocols and used to validate DO measurements from OxyGuard probes and to adjust programmed values to maintain treatment targets when necessary.

Individual tanks were supplied with a 7-color LED aquarium light (Aquaillumination, Hydra 52), programmed to simulate a diel light cycle over a 12:12 h photoperiod, and set to maximum irradiance of ~ 300 µmol photon m^−2^ s^−1^ (PAR). This maximum intensity is likely lower than what corals may experience in situ during peak midday irradiances, however, it matches the irradiance levels these corals were maintained at in the ex situ nursery (320 ± 182 SD, n = 45) and is sufficient to stimulate maximal photosynthesis without causing light stress^45^. Light levels were measured with a light meter (Licor, LI-1400) and an underwater spherical quantum sensor (LI-193SA) submerged at the center of each tank at the start of each experiment and again after five days. Oxygen, temperature, and light conditions were effectively maintained at or near targeted levels for the duration of each experiment (Fig. 1, Table 1), with minimal differences between replicate tanks within a treatment (Tables S1, S2). We did not simultaneously manipulate pH in treatment tanks, which resulted in differences in pH between treatments due to bubbling with nitrogen gas (Tables 1, 2).

### Coral condition

Corals were monitored daily during the experiments for changes in live tissue cover and photophysiology, and then destructively sampled at the end of the experiment for symbiont densities. Fragments were visually evaluated at each time point for evidence of bleaching, tissue loss, and mortality. Tissue loss was quantified as the percent of each fragment with exposed skeleton, resulting from the sloughing of tissue. Fragments were categorized as dead when all living tissue was lost.

The *A. cervicornis* experiment ended after five days, at which point 50% of corals in the lowest DO treatment were dead or displayed partial mortality. We ended the *O. faveolata* experiment after 11 days, more than twice the duration of the *A. cervicornis* experiment (Fig. 1), at which point no observable signs of deterioration were detected in corals from any treatment (Fig. 2a).

### PAM fluorometry

PAM fluorometry is a non-destructive method that directly measures chlorophyll fluorescence and the activity of photosystem II (PSII)^57^, and the quantum yield can be used as a proxy for coral stress^36,58^. For all PAM readings, one measurement was taken from the same position on each fragment with the probe held at a 90º angle ~ 0.5 cm from the coral surface (angle and distance were maintained with a Walz probe holder). To optimize initial fluorescence (F_0_) to between 300–500, the following PAM settings were used: gain = 2, damp = 2, saturation intensity = 8, saturation width = 0.8, and measuring light intensity = 10 for *A. cervicornis* and 6 for *O. faveolata*. PAM measurements were taken pre-dawn daily, at 0500–0600 h. The position on *A. cervicornis* fragments was shifted if tissue sloughing occurred so that measurements were taken on living tissue. After the final PAM measurements, corals were snap frozen in liquid nitrogen and stored at − 80 ºC for subsequent analyses.

### Symbiont density

Symbiont density analyses were conducted at the University of Florida in Gainesville, FL following standard protocols (sensu^59^). In brief, tissue was stripped from the coral skeleton with an air brush (Master Airbrush S68) and filtered seawater. The tissue slurry was then homogenized with a handheld electric tissue homogenizer (Tissue-Tearor) and subsampled for symbiont counts. Symbiont cells were counted with a hemocytometer, with six replicate counts per fragment. Replicate counts were averaged per fragment for all analyses.

Surface area of *A. cervicornis* fragments was determined by wax dipping^60^ and the surface area of *O. faveolata* fragments was determined through image analysis in ImageJ. For *A. cervicornis*, surface area was then corrected by the percent tissue loss recorded for each fragment. Symbiont densities were normalized to fragment live tissue surface area and expressed as cells per cm^−2^.

### Statistical analyses

All analyses were conducted in R (v 4.0.2)^61^. The effects of fixed and random factors were evaluated with linear mixed effects models using the package *lme4*^62^. Normality and homoscedasticity of variances of response variables were evaluated by visual inspection of residuals and Levene’s tests, respectively. All variables met model assumptions, and results are reported for full models that included all fixed and random effects.

For maximum quantum yield and tissue loss, the factor corresponding to measurements repeated daily from the start to end of the experiment (i.e., Day 1, 2, 3, etc.) is referred to as “day”. Day, genotype, and treatment were treated as fixed factors in the analysis of maximum quantum yield and tissue loss, with individual included as a random factor to account for repeated measures, and tank as random factor to account for fragments of different genotypes within a tank (Tables 2, 3). For symbiont density, genotype and treatment were analyzed as fixed factors, and tank as a random factor (Table 4). The significance of fixed effects was evaluated with type II ANOVA tables using Satterthwaite’s method, and Tukey’s post-hoc tests were used where necessary to determine significant differences between levels of a factor using the package *emmeans*^63^. Data in main figures in which genotype was not a significant effect (i.e., all response variables) are presented as treatment averages (± SE), pooled across genotypes.

## Supplementary Information

Supplementary Information.

## Data Availability

The raw data and code for this study are available via Smithsonian Figshare at https://doi.org/10.25573/data.14478252.v3.
