# Supplementary material for: Differential susceptibility of reef-building corals to deoxygenation reveals remarkable hypoxia tolerance
Source: Sci Rep. 2021 Nov 30;11:23168. doi: 10.1038/s41598-021-01078-9 (PMC8632909; doi:10.1038/s41598-021-01078-9)
Supplement: Supplementary file 1 — Supplementary Information. [file 41598_2021_1078_MOESM1_ESM.pdf]

Differential susceptibility of reef-building corals to deoxygenation reveals remarkable hypoxia tolerance

Maggie D. Johnson, Sara D. Swaminathan, Emily N. Nixon, Valerie J. Paul, Andrew H. Altieri

Supplemental Figures

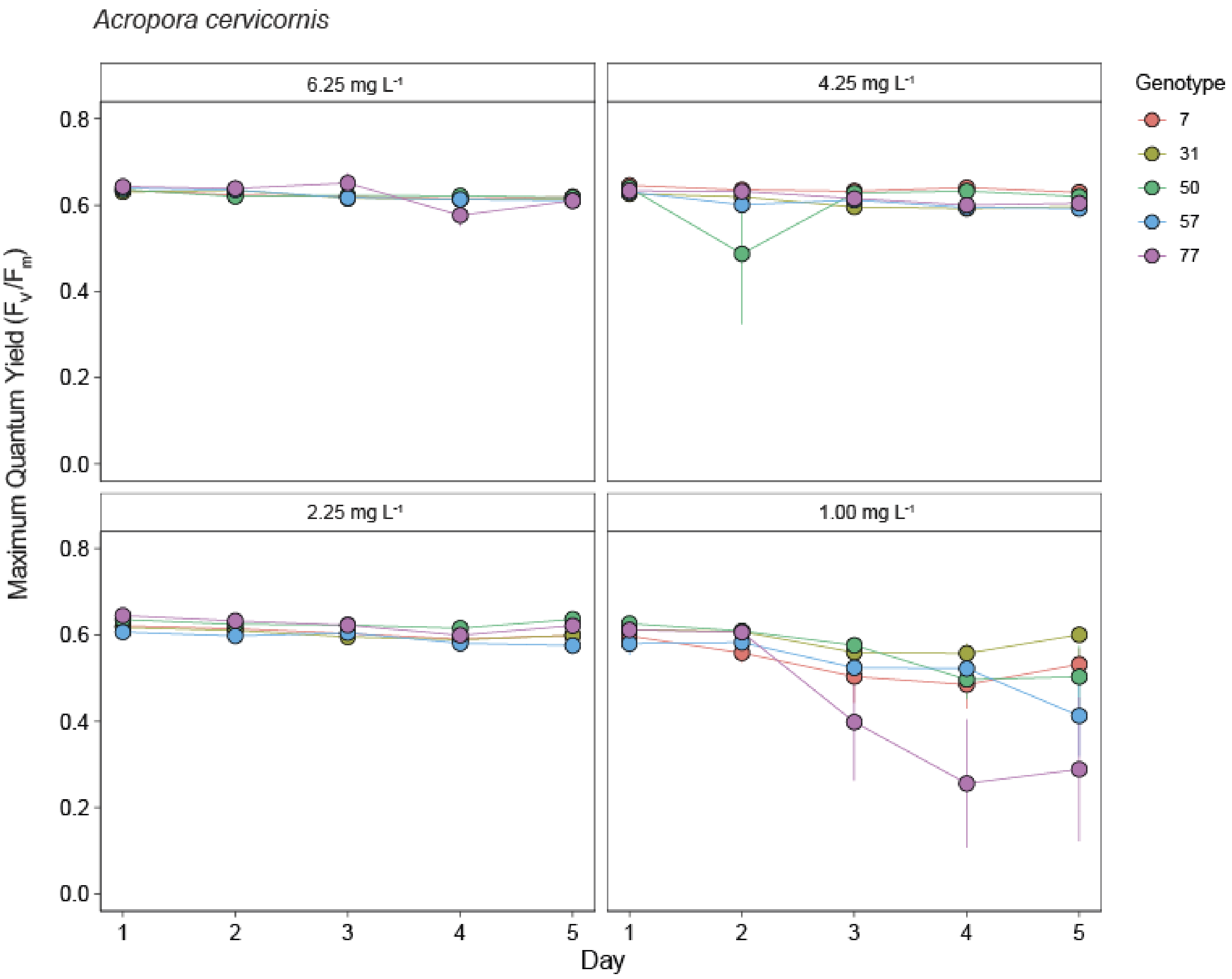

Supplemental Figure 1. *Acropora cervicornis* quantum yield over time by genotype. Mean ± SE maximum quantum yield (F_v/F_m) of *Acropora cervicornis* for the duration of the experiment (n = 3 per genotype). Circles represent daily genotype averages for each treatment, and are color coded by genotype. Panels correspond to different deoxygenation treatments.

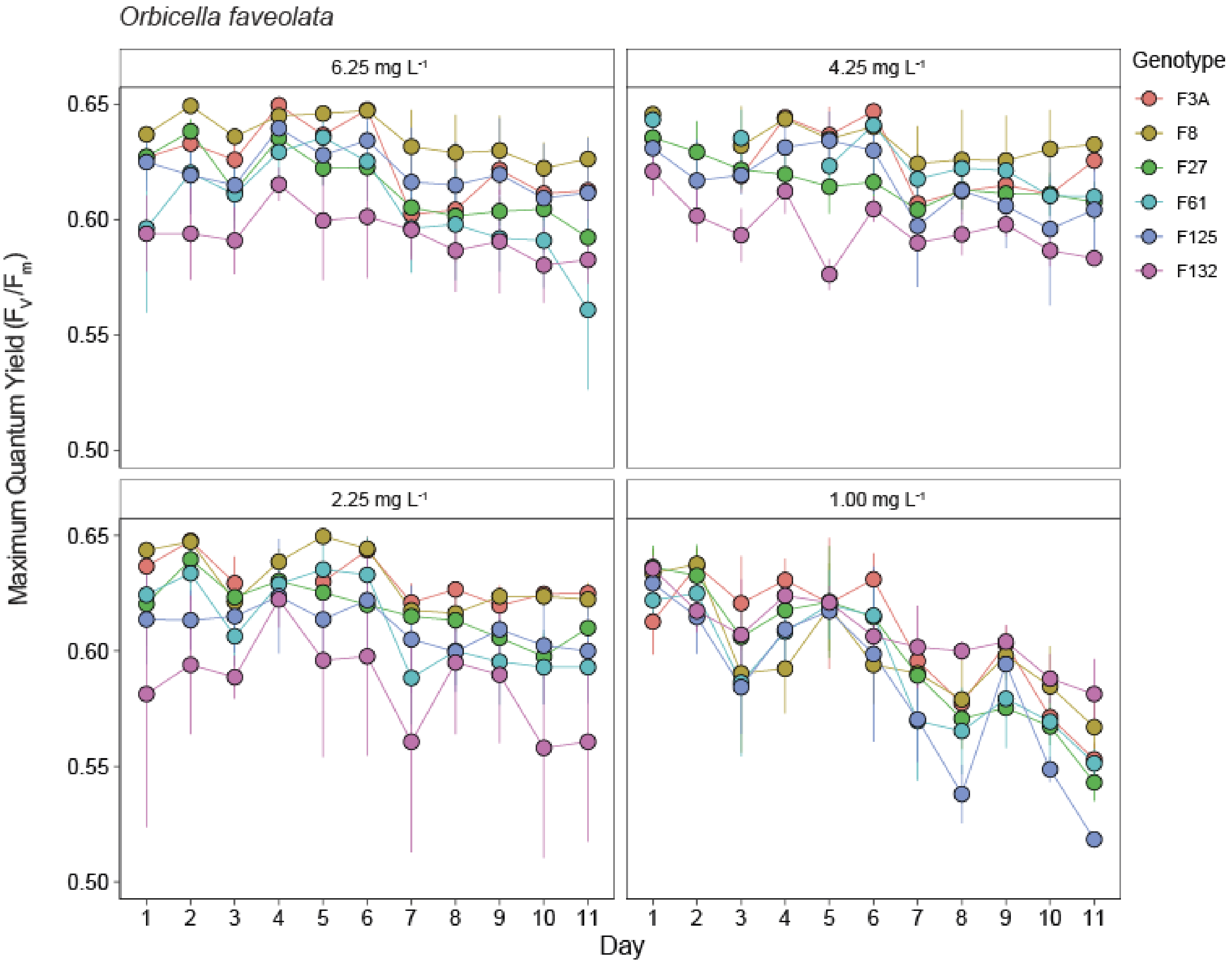

Supplemental Figure 2. *Orbicella faveolata* quantum yield over time by genotype. Mean ± SE maximum quantum yield (F_v/F_m) of *Orbicella faveolata* for the duration of the experiment (n = 3 per genotype). Circles represent daily genotype averages for each treatment, and are color coded by genotype. Panels correspond to different deoxygenation treatments.

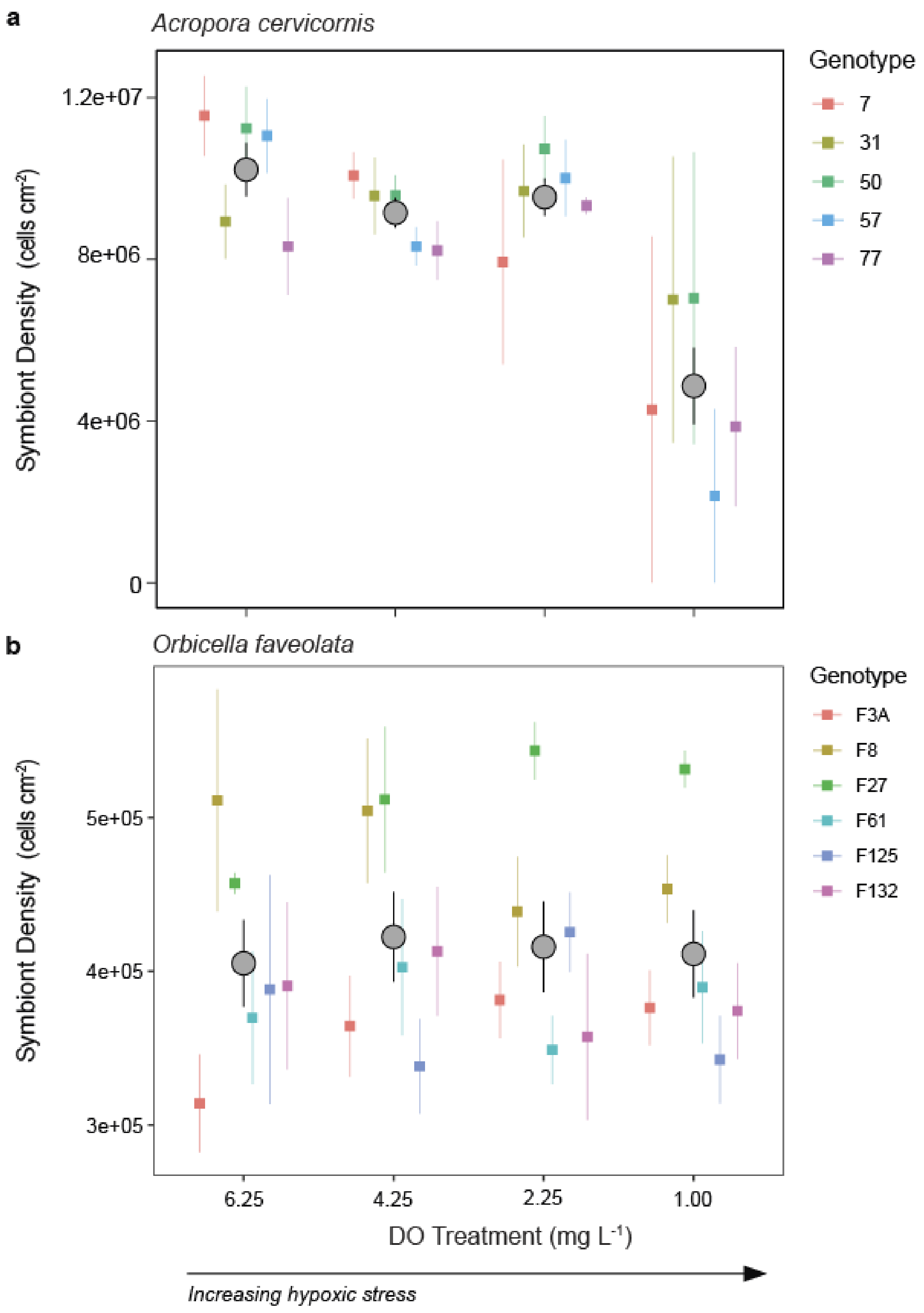

Supplemental Figure 3. Symbiont densities over time by genotype. (a) Mean ± SE symbiont densities of (a) *Acropora cervicornis* and (b) *Orbicella faveolata* (n = 3 per genotype, n = 3 per treatment). Grey circles represent treatment means across all genotypes, and colored squares represent the mean of each genotype within a treatment.

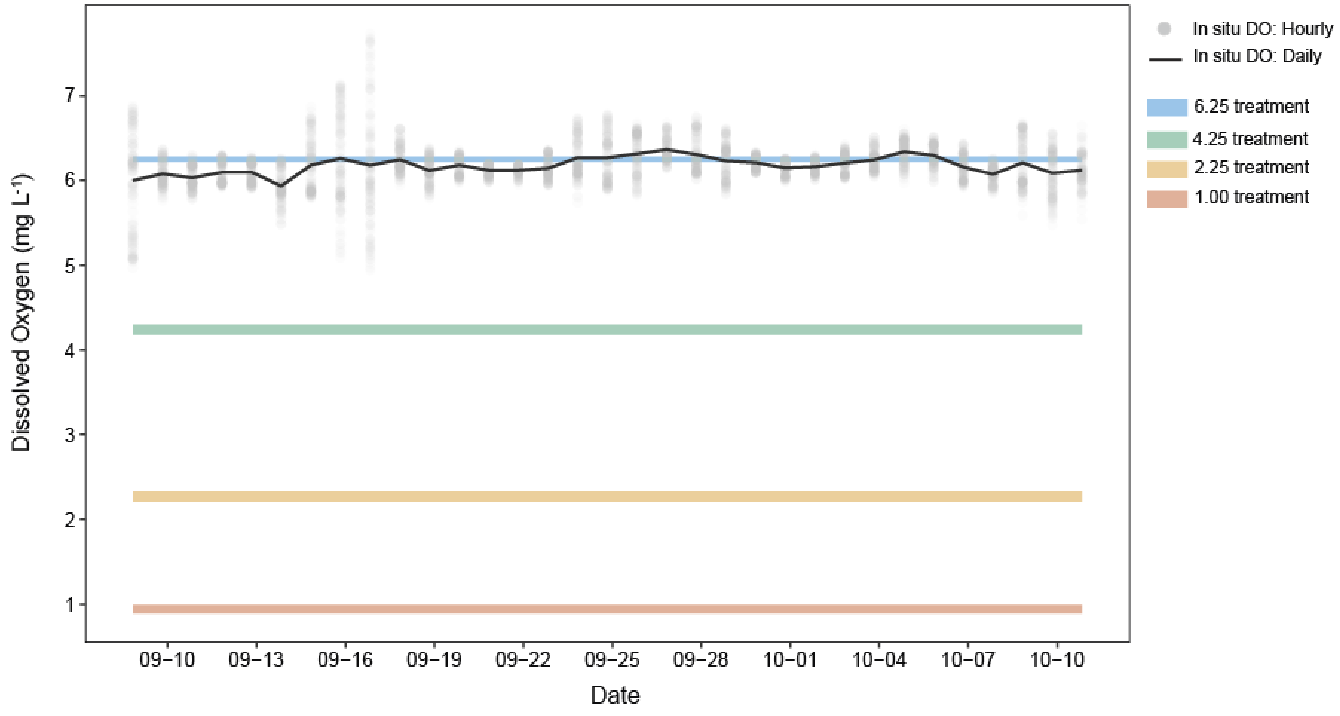

Supplemental Figure 4. In situ and laboratory dissolved oxygen conditions. Daily average dissolved oxygen (DO) concentrations at the Mote Marine Laboratory in situ coral nursery (black line), with hourly average DO values represented by grey circles. The laboratory deoxygenation treatments are overlaid as solid rectangles, with the center of the rectangle at the mean DO concentration and the height of the rectangle ± SD for the respective treatments.

Supplemental Tables

Table S1. Mean (SD) physical parameters from daily discrete measurements. Data were averaged by tank for the duration of the experiment (n = 13 days). pH is on the NBS scale, salinity is in practical salinity units, and PAR is in µmol photons m^{-2} s^{-1}.

Species	Treatment	Tank	DO (mg L^{-1})	Temp (°C)	pH	Salinity	PAR
O. faveolata	6.25	2	6.18 (0.05)	26.6 (0.04)	8.00 (0.04)	35.12 (0.14)	292 (20)
		5	6.30 (0.09)	26.6 (0.05)	7.95 (0.06)	35.07 (0.09)	301 (7)
		9	6.28 (0.12)	26.6 (0.05)	8.00 (0.07)	35.16 (0.28)	290 (25)
	4.25	4	4.24 (0.04)	26.6 (0.05)	8.05 (0.04)	35.05 (0.07)	286 (7)
		7	4.42 (0.05)	26.5 (0.03)	7.94 (0.08)	35.06 (0.17)	296 (5)
		11	4.08 (0.09)	26.7 (0.04)	8.03 (0.04)	35.10 (0.10)	349 (66)
	2.25	1	2.24 (0.04)	26.5 (0.05)	8.12 (0.05)	35.07 (0.11)	302 (61)
		8	2.31 (0.03)	26.6 (0.05)	8.07 (0.04)	35.07 (0.13)	297 (71)
		12	2.25 (0.05)	26.6 (0.05)	8.00 (0.05)	35.06 (0.10)	337 (48)
	1.00	3	0.95 (0.08)	26.6 (0.03)	8.32 (0.04)	35.06 (0.08)	289 (15)
		6	0.96 (0.07)	26.6 (0.03)	8.16 (0.08)	35.10 (0.08)	298 (2)
		10	0.92 (0.06)	26.6 (0.05)	8.24 (0.07)	35.08 (0.11)	302 (27)

Table S2. Mean (SD) physical parameters from daily discrete measurements. Data were averaged by tank over the duration of the experiment (n = 4 days). PAR was measured on one day. pH is on the NBS scale, salinity is in practical salinity units, and PAR is in µmol photons $m^{-2} s^{-1}$.

Species	Treatment	Tank	DO (mg L^{-1})	Temp (ºC)	pH	Salinity	PAR
A. cervicornis	6.25	3	6.31 (0.07)	26.4 (0.22)	7.91 (0.03)	35.31 (0.20)	315
		8	6.35 (0.16)	26.3 (0.25)	7.89 (0.03)	35.10 (0.10)	318
		9	6.47 (0.08)	26.4 (0.13)	7.94 (0.04)	35.20 (0.18)	323
	4.25	2	4.24 (0.10)	26.4 (0.10)	7.78 (0.05)	35.20 (0.20)	319
		5	4.25 (0.04)	26.4 (0.16)	7.96 (0.09)	35.13 (0.14)	319
		7	4.31 (0.10)	26.3 (0.17)	7.85 (0.07)	35.27 (0.24)	314
	2.25	1	2.37 (0.09)	26.1 (0.19)	8.00 (0.03)	35.21 (0.21)	310
		10	2.38 (0.10)	26.4 (0.26)	7.99 (0.04)	35.21 (0.19)	310
		12	2.24 (0.03)	26.1 (0.08)	7.96 (0.09)	35.24 (0.17)	316
	1.00	4	0.84 (0.11)	26.5 (0.91)	8.04 (0.10)	35.25 (0.25)	313
		6	0.85 (0.07)	26.2 (0.21)	8.14 (0.04)	35.47 (0.43)	307
		11	0.91 (0.20)	26.2 (0.10)	8.15 (0.06)	35.34 (0.26)	309

Table S3. Mean ± SD physical parameters from discrete measurements of acclimation (i.e., holding) tanks. Corals were maintained in holding tanks for 4-6 weeks prior to experimentation. Conditions were measured every 3-4 days (n = number of measurements per species). pH is on the NBS scale, salinity is in practical salinity units.

**Species**	**DO (mg L^{-1})**	**Temp (ºC)**	**pH**	**Sal**
O. faveolata (n = 17)	6.53 ± 0.30	26.73 ± 0.14	8.08 ± 0.04	35.38 ± 0.54
A. cervicornis (n = 19)	6.77 ± 0.10	27.62 ± 0.24	8.00 ± 0.05	35.37 ± 0.34
